# The importance of illumination in nest site choice and nest characteristics of cavity nesting birds

**DOI:** 10.1038/s41598-017-01430-y

**Published:** 2017-05-02

**Authors:** Paweł Podkowa, Adrian Surmacki

**Affiliations:** Department of Avian Biology and Ecology, Faculty of Biology, Adam Mickiewicz University, Umultowska 89, 61-614 Poznań, Poland

## Abstract

Light has a significant impact on many aspects of avian biology, physiology and behaviour. An increasing number of studies show that illumination may positively influences birds’ offspring fitness by e.g. acceleration of embryo development, stimulation of skeleton growth or regulation of circadian rhythm. Because nest cavities have especially low illumination, suitable light levels may be especially important for species which nest there. We may therefore expect that birds breeding in relatively dim conditions should prefer brighter nest sites and/or evolve behavioral mechanisms to secure sufficient light levels in the nest. Using nest boxes with modified internal illumination, we experimentally tested whether light regime is a cue for nest site selection of secondary cavity-nesting species. Additionally, we investigated whether nest building strategies are tuned to internal illumination. Our results demonstrate that, nest boxes with elevated illumination were chosen twice as often as dark nest boxes. Moreover, birds built higher nests in dark nest boxes than birds in boxes with elevated illumination, which suggests a mechanism of compensating for low light conditions. Our results provide the first experimental support for the idea that nest site choice and nest building behaviour in cavity-nesting birds are influenced by ambient illumination.

## Introduction

The majority of bird species are diurnal and thus experience changes in light intensities associated with daily, lunar or seasonal cycles. Not surprisingly, light has an immense impact on many aspects of avian physiology, biology and ecology. In birds, three organs (eyes, pineal organ and hypothalamus) regulate photoperiodism and circadian rhythms^1, 2^. The orientation of the avian magnetic compass depends on interactions between blue light and photoactive molecules in the retina^3^. Illumination timing affects duration of singing, development of the reproductive system, testosterone levels and moult in male birds^4, 5^. Similarly, illumination timing and light spectrum regulates the hormonal control of fecundity in females; a mechanism used by man for decades to maximize egg production in domestic hen (e.g. *Gallus domesticus* ^6–9^), but also observed in wild birds^10^.

A recent study^11^ has shown that in many bird species eggshells are transparent enough for light to reach the photoreceptors of developing embryos. Moreover, for the majority of species, illumination levels at the nest and incubation behavior of adults (so called off-bouts) do not eliminate light completely^12^. According to a recent review^13^, an exposure to light during incubation may positively affect various aspects of bird physiology including: thermo-regulation, photo-acceleration of embryos development, lateralization, circadian rhythm, DNA repair through photo-reactivation and antimicrobial defense. Thus far, most of the above hypotheses have not been rigorously tested, especially in wild birds. However, there is experimental evidence of photo-acceleration of embryo development for both wild species^14, 15^ and the domestic hen^16–20^. Light may also be beneficial for nestling growth and health. For example, domestic chicks exposed to constant light gain significantly more weight compared with controls reared on a 12L:12D light cycle^21^. Light of the shortest wavelengths (UV) may also play a crucial role in normalizing bone growth in chicks by stimulating the synthesis of vitamin D_3_^22^. Finally, light conditions at the nest site may also affect brood rearing behaviour of adult birds. For example, in cavity nesting species, the minimum required amount of light seems to be necessary for birds to see and feed their young efficiently^23, 24^. Although recent studies have shown positive influence of light on birds breeding biology, its negative effect (e.g. in sleep disruption by artificial light) has been also reported^25, 26^.

Considering the potentially beneficial effects of light on avian embryo development, nestling condition and adults’ behaviour, a variety of mechanisms that could improve nest light environment could evolve. It has been demonstrated that eggshell pigmentation modifies its transparency depending on ambient light spectrum and intensity. Eggshells of species living in darker environments tend to be less pigmented compared to eggshells of species that nest in open habitats to compensate for the light deficiency^11^. Another mechanism that can enhance egg illumination is incubation off-bouts when clutches usually remain uncovered for short periods of time^27^. The main function of this behavior is to forage^27^ however, another non-exclusive explanation is that off-bouts provide light for developing embryos^12^. In cavity nesting species, where light intensity is very low, the height of the nest layer may improve illumination, as it can be negatively correlated with a cavity depth^23, 24, 28^. Finally, parents may choose among available nest sites those sites that provide the most favorable light conditions for developing offspring^24^.

Despite a growing body of evidence of the importance of light for the condition of avian embryos and nestlings, there are virtually no studies testing the role of light on nest site selection in birds. The only exception is an experimental study performed on domestic hens in artificial laboratory conditions^29^. In wild living species, there are only a few observational studies of nest box breeding birds, from which no solid conclusions can be drawn^30–32^ (see details in Discussion). Marked preferences for light nest sites should be especially pronounced in cavity-nesting species, which experience very low intensity of light, especially in a deep cavities^23, 24^.

This study has twofold goals. The first is to test experimentally whether light intensity has an effect on nest site selection. We used a secondary cavity-nesting species, the great tit (*Parus major*) breeding in nest boxes with modified interior illumination. Considering a general positive effect of light on nestling condition and adults’ behaviour, we predicted that nest boxes with elevated brightness would be occupied more often and earlier in the season, comparing to control, dark analogues. The second goal of the study was to test how light conditions affects nest building behaviour of adult birds. We predicted that in standard, dark nest boxes birds will build higher nest comparing to bright nest boxes, which is a mechanism that regulates the amount of interior light.

## Results

### Illumination inside nest boxes

During the nest box occupation period (between 5 and 15 April), both the light transfer and the illumination were lower in dark nest boxes (Me = 0.02%, Q_25–75%_ = 0.01–0.03; Me = 1.41 lx, Q_25–75%_ = 1.04–2.19, respectively), compared to bright nest boxes (Me = 0.88%, Q_25–75%_ = 0.42–1.11; Me = 52.67 lx, Q_25–75%_ = 42.83–62.28, respectively). The differences were strongly statistically significant, both for light transfer (Mann-Whitney *U* test; Z = −8.393, n = 95, p < 0.001) and illumination (Mann-Whitney *U* test; Z = −8.393, n = 95, p < 0.001).

**Figure 1:**
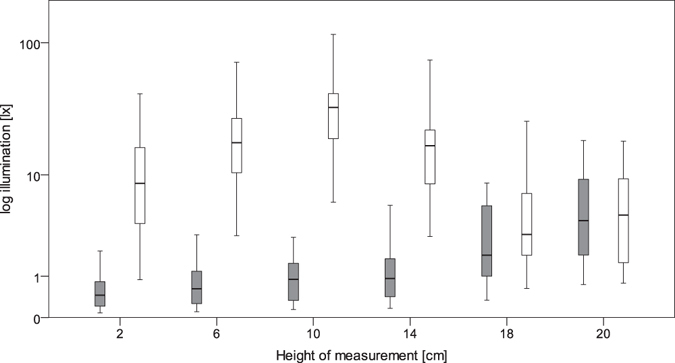
Illumination at different distances from the base of the nest box. Explanations: grey - dark nest boxes; white - bright nest boxes.

The height above the nest box floor had a significant effect on internal illumination in both bright (Friedman test, Q = 67.267, df = 5, p < 0.001) and dark nest boxes (Friedman test, Q = 69.590, df = 5, p < 0.001; Fig. 1). The detailed results of multiple pairwise comparisons using Nemenyi’s procedure are shown in Table 1. In dark nest boxes, there was a gradual increase of illumination (Fig. 1) and light transfer between 2 and 20 cm above the nest box floor. In bright nest boxes, there was an increase in illumination reaching the highest value at 10 cm followed by a decline to 18 cm and a small increase at 20 cm (Fig. 1).

**Table 1:** Pairwise differences in illumination at different distance from the nest box floor in brightened and dark nest boxes. Results shown are significance levels (*p*) of Nemenyi’s post hoc tests.

Type of nest box	Distance from the nest box floor	6	10	14	18	20
Bright	2	n/s	**<0.01**	n/s	n/s	n/s
Dark		n/s	n/s	**<0.01**	**<0.01**	**<0.01**
Bright	6		n/s	n/s	**<0.01**	**<0.01**
Dark			n/s	n/s	**<0.01**	**<0.01**
Bright	10			n/s	**<0.01**	**<0.01**
Dark				n/s	**<0.05**	**<0.01**
Bright	14				**<0.01**	**<0.05**
Dark					n/s	**<0.05**
Bright	18					n/s
Dark						n/s

### Nest site choice

**Figure 2:**
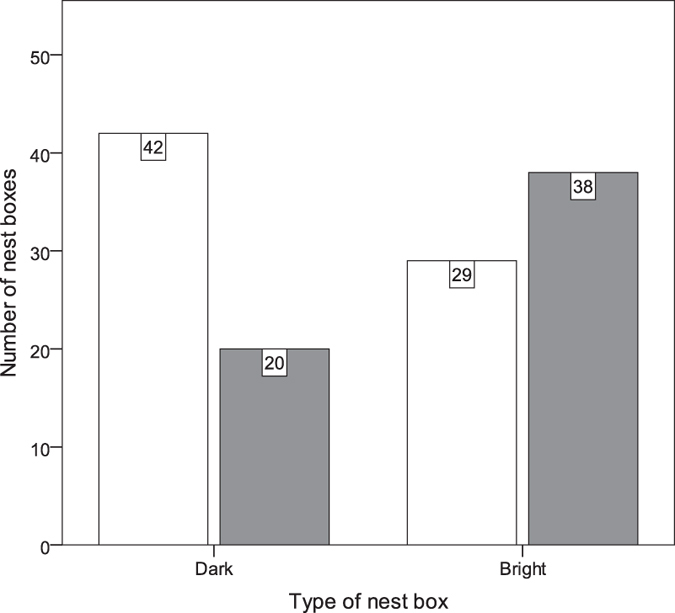
Nest box occupation by great tits in relation to light conditions. Grey bars – occupied, white bars – unoccupied.

The results of the logistic regression showed a significant effect of nest box internal illumination (nest box type), previous-year occupation, and study site on nest box choice (results shown in Table 2). However, the most important effect was the nest box type, which was indicated by the highest odds ratio (OR, Table 2). The proportion of bright and dark nest boxes occupied by great tit was 1.9:1 (Fig. 2). Both study sites II and III were more attractive for individuals than study site I. Moreover the interaction between study site and nest box type was not significant, which means that nest box choice was not linked to study site. The effect of previous-year occupation was also statistically significant but its’ odds ratio was lower than the other significant factors. We performed two goodness of fit tests supporting our logistic regression model: Omnibus Tests of Model Coefficients: χ²_(6)_ = 28.73; p < 0.001 and Hosmer & Lemeshow χ²_(7)_ = 2.23; p = 0.946.

**Table 2:** Logistic regression model assessing predictors of nest box occupation by great tits in 2016. Study site (1) and (2) refers to dummy variables (see Methods).

Predictor	β	SEβ	Wald’s	df	p	OR (95% CI)
Nest box type	1.864	0.61	9.348	1	**0.002**	6.45 (1.95–21.3)
Occupied in 2015	1.087	0.425	6.533	1	**0.011**	2.97 (1.29–6.82)
Study site	0	0	7.696	2	**0.021**	
Study site (1)	1.756	0.704	6.221	1	**0.013**	5.79 (1.46–23)
Study site (2)	1.733	0.796	4.743	1	**0.029**	5.66 (1.19–26.94)
Study site* Nest box type			2.322	2	0.313	
Study site (1)* Nest box type	−1.347	0.972	1.923	1	0.165	0.26 (0.04–1.75)
Study site (2)* Nest box type	−1.155	1.065	1.177	1	0.278	0.32 (0.04–2.54)
Intercept	−2.341	0.561	17.397	1	**0.000**	0.10

The second measure of nest site preference was the sequence of nest boxes occupancy expressed by the first egg laying date (Table 3). We did not find any effect of the nest box internal illumination (Table 3). However, the result of the GLM showed a significant effect of study site on the nest box occupation sequence pattern (Table 3). There was a weakly significant trend towards nest boxes at site II being occupied significantly earlier comparing to the site I (Tukey’s *post hoc* test, p = 0. 049).

**Table 3:** Results of GLMs explaining variation in nest box occupation sequence and nest height.

	Explanatory variables	Mean Square	F	df	p
Occupation sequence	Nest box type	25.48	1.59	1	0.216
	Study site	81.82	5.09	2	**0.011**
	Occupied in 2015	3.07	0.19	1	0.664
Nest height	Nest box type	247.07	34.79	1	**<0.001**
	Study site	6.58	0.93	2	0.406
	Clutch size	15.28	2.15	1	0.151
	Egg laying date	11.48	1.62	1	0.212

### Nest height

**Figure 3:**
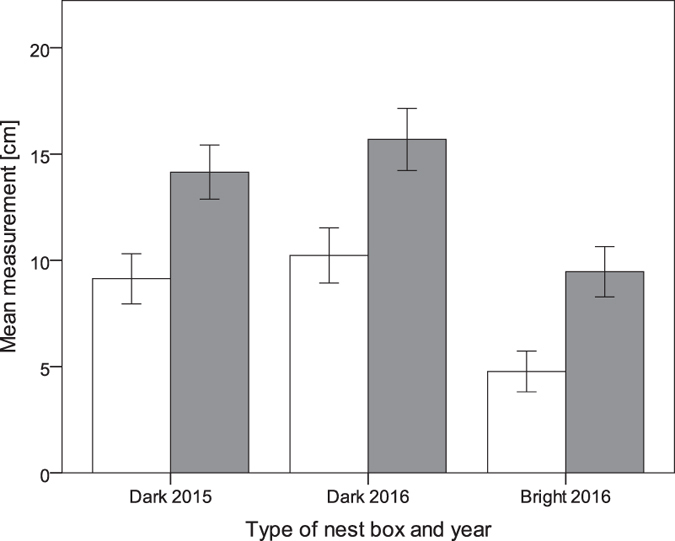
The mean nest height (white bars) and nest depth (grey bars) in dark and bright nest boxes, data collected in 2015 and 2016. Error bars shows 95% confidence intervals.

The mean ( ± SD) height of the nest bottom was significantly greater in dark (10.23 cm ± 2.44) than in bright boxes (4.78 cm ± 2.66; Table 3, Fig. 3). Among the tested factors, only nest box type significantly influenced nest height, while the effect of egg laying date, study site and clutch size remained not significant (Table 3).

## Discussion

The results presented in this study provide the first known experimental evidence for the importance of light in the nest environment of a wild-living avian species. Great tits chose nest boxes with artificially elevated illumination almost twice as often compared to standard dark nest boxes. Beside of nest site choice, light inside nest boxes affected also the nest building strategy. Great tits tuned the nest height to gain the greatest illumination for the nest-cup contents in a given nest box type.

Existing data on avian preferences to occupy well illuminated nests sites are sparse and based mainly on observational studies. The only experimental survey was performed on domestic hens^29^. Birds could choose between dark and artificially illuminated nest boxes to lay eggs. The main assumption of that study was that hens prefer dark sites because lower illumination is evolutionary associated with more concealed and thus safer nest sites. However, only one of four experimental groups of hens followed this pattern. Moreover, preferences for nest sites with lower illumination depended also on the maturity and earlier experience of birds. In another study^32^, nest box use by American kestrels (*Falco sparverius*) depended on internal illumination during the nestling period but not during the nest site selection period. This result suggested some importance of light in nest-site choice, but due to the observational nature of the study, they could also be a product of a correlation between illumination and other habitat variables, e.g. the degree of nest box concealment by vegetation^32^. In a number of studies, bird preferences for interior coloration of nest boxes have been observed. Nest boxes painted white are favored compared to those painted black or unpainted in the following species: common goldeneye (*Bucephala clangula)* ^31^, pied flycatcher (*Ficedula hypoleuca)* ^33^, eastern bluebird (*Sialia sialis*) and house sparrow (*Passer domesticus)* ^34^. In contrast, starlings (*Sturnus vulgaris*) show high preference for black interiors^30^, whereas for tree swallows (*Tachycineta bicolor*) and house wrens (*Troglodytes aedon*), no evident pattern has been reported^35^. Those findings could be interpreted as a preference for brighter nesting conditions because light reflected from white walls increases overall internal illumination^33^. On the other hand, those experimental designs do not allow the separation of the effect of light from the effect of walls’ coloration or presence of paint. The importance of light conditions on nest site choice in secondary cavity-nesters breeding in natural conditions was investigated in Białowieża primeval forest (E Poland)^23, 24^. These findings demonstrate that mesopic-scotopic range of illumination that occurs in nest-holes is close or below a threshold enabling color vision^36^. Thus, many tree cavities may be too dark to be usable by birds, which might explain their underutilization by birds^24^. This assumption is indirectly supported by the fact that nests of collared flycatchers (*Ficedula albicollis*) in cavities with entrances oriented upward (and potentially entering more light), were located deeper compared to cavities with entrance oriented sideward^24^.

Nest-site selection in birds is a complex decision-making process, which involves many factors of varying importance (e.g. food availability, nest site availability and social environment^37^). In the studied population, beside the nest box illumination, these were previous year nest box occupancy and habitat type. These findings are consistent with previous studies suggesting, that birds assess nest site attractiveness using evidence of successful breeding left by former residents or themselves the previous year^38^. Habitat preferences for sites dominated by oak (*Quercus petrea Liebl*.) have previously been observed in other great tit populations^39^ and are most likely to be an effect of better foraging conditions. Oak is the most important host-plant for caterpillars^40^, which are a crucial component of great tit nestling diet^41^. Habitat preferences were mirrored by the sequence of nest box occupancy found in our population. We observed the earliest egg laying in oak-dominated sites. In many bird species, including great tits^39^, the sequence of occupancy of breeding areas depends on quality of habitat patches. The best habitats are occupied first, followed by suboptimal and marginal ones^37^. Contrary to our expectations, brightened nest boxes were not occupied earlier than dark ones. The most plausible explanation of this pattern is that brightened nest boxes were not limited in the study area. Great tits occupied less than a half of the available nest boxes and 29 brightened nest boxes were left unoccupied by any bird species. This suggests that preferred nest sites were not limited in the study area, so they were not likely be an object of severe competition. Another explanation is that nest box (territory) occupation took place long before nest building and is not correlated with the timing of reproduction. On the other hand, the date of egg laying may be determined by food availability which was equal for bright and dark nest boxes.

Great tits that chose dark nest boxes built significantly deeper nests compared to birds breeding in brightened nest boxes. Several lines of evidence suggest that this relationship was shaped by preferences for light. First, illumination inside dark nest boxes showed an increasing trend from the nest box bottom to the entrance hole. This correlation is intuitive, as the entrance hole is the only source of light in cavities (compare^23, 24^). Thus, selection for building higher nests appears to be the only mechanism that enables improvement of light conditions in the dark, unmodified cavities. On the contrary, in brightened nest boxes, light remained at very high level (>10 lux) across the entire depth of the nest box. In such conditions birds do not need to build high nests to obtain the desired illumination. Because nest building is a condition-dependent behavior^42–44^, we may therefore expect, that in the absence of other selective pressures, birds build shallower nest to save time and energy for other activities. Another advantage of building shallower nests is to decrease predation risk by increasing the distance between nest contents and the entrance hole^45^. Our results provide the first experimental evidence that cavity-nesting species actively manipulate nest height to achieve the desirable amount of light in the nest cup. Great tits, and many other cavity-nesting passerines, built nests of a greater height when compared to other similar sized open-nesters. Moreover, many studies report great variability of their nest dimensions, even in nest boxes^46^. To date, no explicit explanation for selective pressures for building deeper nests in cavities have been found. It has been suggested that deeper nests provide protection from soaking^47^ or plays a role in sanitation^48^. However, unlike horizontal dimensions^49, 50^, nest height is not related to various parameters of breeding success^46, 49, 51, 52^ (but see ref. 53 for a positive relationship between the nest depth and breeding success in blue tits). On the other hand, building big nests is a costly and condition-dependent process, which has been demonstrated for tits^42–44^ and other bird species (reviewed^54^). The costs of building larger nests encompass not only greater time and energy expenditures^54^, but also longer exposure of adults for predators^55^. Our results show that illumination may be one of the most important, and generally overlooked factors, causing selection for higher nests of cavity-nesting species.

Most of the previous studies focused on the role of nest predation as the main force reducing nest height in cavity nesting passerines. Studies in natural conditions demonstrate that there is a minimal distance between the entrance hole and the nest-cup which prevents terrestrial predators like pine martens (*Martens martens*) from reaching the nest content with their paws (so called “danger distance”^45^). To obtain the desired greater entrance-nest-cup distance, birds may choose deeper cavities, build shallower nests or both. Experimental and observational studies seem to confirm this mechanism. Great tits that bred in taller nest boxes (with longer entrance-floor distance) built deeper nests compared to shallower nest boxes^56^. The same pattern was reported from a great tit population breeding in natural nest holes^28^. Similarly, the nests of great and blue tits breeding in nest boxes fitted with anti-predator entrance tubes were higher compared to a control nest boxes with standard entrances^57^. However, it is important to notice that cavity (or nest box) depth affects both light conditions and predation risk, so the effect of both factors on the nest depth might be difficult to untangle. We therefore cannot rule out the possibility that greater nest height in predator-safe nest boxes at least partially an effect of compensating for low light in deep cavities.

Although illumination had an overwhelming effect on nest depth in our population, in most cases it is a result of a trade-off between different conflicting selection pressures, e.g. predation and light. For example, nesting further from the entrance would give collared flycatchers a selective advantage due to predation avoidance^24^. Despite that, many flycatchers build nests relatively close to the entrance which makes them accessible to pine martens, but on the other hand, probably also provides the minimal amount of light for the brood^24^. In our study, the mean nest height in dark nest boxes (ca. 10 cm) was below the greatest illumination level (ca. 20 cm). This result suggests that both illumination and predation avoidance may influence the height the bottom of nests of great tits.

Our results imply an important question: why have preferences for light in the nest environment evolved? Although there are a number of hypotheses pointing at possible advantages of exposing the clutch and/or nestlings to the light^13^, experimental studies testing these in wild birds are rare. Laboratory experiments on house sparrows^14^ and blackcaps (*Sylvia atricapilla*)^15^ showed that longer exposure of eggs to light increase the rate of embryonic development and confirms the photo-acceleration hypothesis previously tested on poultry. It is important to notice that the response to light in blackcaps was weaker than in house sparrows, which could result from higher light-sensitivity of the cavity-nesting species^15^. There are some premises that better illumination at the nest site may be beneficial for parents by improving their ability to assess nestling visual begging signals (i.e. mouth coloration)^58^, discrimination between their own and parasitic eggs^59, 60^ or to aid in general orientation in dim cavities^23, 24^. Future surveys are needed to investigate the possible fitness consequences of light within nest sites of birds. Such studies should embrace both physiological effects of light on early stages of nestling development (e.g. growth rate, condition, functional lateralization) and light-dependent effects on adults’ behavior (e.g. incubation, feeding strategies).

Our study opens new avenues for further research on light-dependent nest site selection in birds. Further studies are needed to investigate whether light preferences are consistent within different ecological groups of birds. We may expect that light might be less important in open-nesting species due to higher overall illumination and selection may even favor behaviors that limit access of light to eggs and nestlings (see ref. 13). On the other hand, even some cavity-nesting species may avoid bright sites^30, 34^ if they are perceived as less secure (compare^29^). The separation of effects of illumination and nest concealment will be one of the most challenging methodological problems in future studies on the importance of light on nest-site selection.

## Methods

### Study area and nest boxes

The study was conducted on a population of great tits breeding in nest boxes in Wielkopolski National Park, Western Poland, in 2016. In 2014, nest boxes (n = 159) were placed at regular 50 meter intervals on 3 study sites (I–III), in a total area 48,35 ha. The number of boxes differed between study sites: I – 89, II – 42, III – 28. Every box was hung at a height of three meters with the entrance hole oriented South-East to standardize light conditions during the day. All the nest boxes had the same size, with internal dimensions of 12.0 × 16.0 × 36.0 cm and the entrance with diameter of 3.3 cm located 24 cm from the floor. To facilitate access to the nest, every nest box was equipped with a “drawer” (12.0 × 16.0 × 12.0 cm), where nests were built. All nest boxes were fitted with two 5.0 cm diameter resin windows (www.Handykam.com®) located in side-walls (one on each side), 14 cm from the bottom. The windows were opaque at the degree that prevents seeing interior of the nest box and transparent enough to let the light in. Each window was equipped with an adjustable shutters made of black plastic sheets.

### General procedures

From the middle of March 2016 onwards, nest boxes were checked regularly to determine the date on which the first egg was laid and to quantify clutch size. We quantified nest size by using two variables: the depth of nest^61^, which was an average of the maximum height of the nest material measured in four corners of the nest box and the height of nest, which was a distance from the nest box floor to the deepest point of the nest-cup bottom. Measurements were performed using a ruler (to an accuracy of 5 mm) and caliper (to an accuracy of 1 mm), respectively. We measured in total 48 nests at the end of the egg laying stage. Both measurements were highly and positively correlated (Pearson: r = 0.97, p < 0.01, n = 48), so in a further analysis we used only nest height. The same nest box monitoring and nest measurements were performed in 2015 when no internal nest box illuminance was modified (i.e. all nest boxes were naturally dark). This data set was used as additional control group (beside dark nest boxes in 2016) to analyze the effect of nest box internal illumination on nest dimensions. Moreover, nest box occupation data collected in 2015 was used as explanatory factor in nest site choice analysis in 2016, because of its potential influence on nest box attractiveness (see ref. 38).

### Experiment design

To test the effect of nest box illumination on nest site choice and nest building behavior, we manipulated illumination inside nest boxes. Between September 2015 and early March 2016, shutters in all nest boxes remained closed. At the beginning of nest site choice period (16–17 March 2016), windows were opened in every second box creating two types of nest boxes: “bright” – with opened shutters (n = 79) and “dark” – with closed shutters (n = 80). In addition to great tits, three other species bred in the nest boxes: pied flycatchers (n = 2), blue tits (n = 16) and European nuthatches (*Sitta europaea*, n = 12). These nest boxes were excluded from further analysis; therefore the final number of bright and dark nest boxes was 67 and 62, respectively. Opening shutters significantly increased both absolute illuminance and light transfer in bright nest boxes comparing to dark nest boxes (see Results). The old nest material was removed from all nest boxes in the autumn preceding the experiment.

Although all nest boxes used in the study were identical with respect to the material used, dimensions and all construction details, opening windows could potentially increase internal temperature, what in turn could influence nest box choice. Therefore, we set temperature loggers (i-Button, Maxim Integrated®) in 91 randomly selected nest boxes (47 dark and 44 bright). They were located on the internal side of the front wall, about 5 cm below entrance hole. Temperature measurements were taken every 30 minutes, from 18 March to 10 April 2016. The time of measurements falls into the limits of nest choice period in great tits in the studied population, which spans between mid-March and mid-April. There were no significant differences in the mean (±SD) day temperature between both groups (dark nest box: 8.39 °C ± 0.28; bright nest box: 8.38 °C ± 0.33; t = 0.096, df = 89, p = 0.924), therefore we did not include temperature in the nest site choice analysis.

### Light measurements

Two measurements of illumination inside nest boxes were performed. The first one took place within the time of nest site choice, between 5 and 15 April 2016. We measured randomly chosen 46 dark and 49 bright nest boxes with no visible evidence of nest material. The purpose of this measurement was to assess the efficiency of opening windows in bright nest boxes comparing to dark nest boxes. The illumination was measured about 2 cm above the nest box floor. The second measurement was performed between 6 May and 8 June, which corresponds to the time of incubation in the studied population. In order to prevent disturbance to broods, we used only nest boxes unoccupied by great tits. We measured 16 randomly chosen nest boxes. The purpose of this measurement was to assess how illumination inside nest box depends on the distance from the nest box floor. We measured illumination 2, 6, 10, 14, 18, 20 cm above the floor. The first measurement was done by putting the lux meter head (of 2 cm height) on the nest box floor. To obtain other heights, we put lux meter head on 4, 8, 12, 16 and 18 cm high cartoon boxes. In the case of two last measurements (18 and 20 cm), windows were partially and completely covered by cartoon boxes, respectively. In each nest box, all measurements were performed twice: with open and shut windows.

Measurements inside nest boxes were performed with a lux meter pointed toward the nest box ceiling. All the measurements per given nest box/distance from nest box floor were taken three times and then averaged. To assess precision of the light measurements, we calculated repeatability^62^ within three measurements taken at the nest box floor in dark and bright nest boxes during the nest site choice period. Analyses show that our measurements were highly and significantly repeatable in both bright and dark nest boxes (r > 0.99, ANOVA, F_48, 98_ = 217, p < 0.01; r > 0.99; ANOVA; F_45, 98_ = 94.72, p < 0.01, respectively). Repeatability of measurements performed during the egg incubation period at six heights in bright and dark nest boxes was also significantly high (r > 0.99 in all cases). Measurements were performed during constant weather conditions (sunny, clear sky), between 08:00 AM and 02:00 PM. Time of measurement may affect brightness level inside nest boxes due to changes of the sun’s position. To cope with this problem, we measured bright and dark nest boxes alternately. There was no significant difference in the time of light measurement between dark and bright nest boxes measured during the time of nest site choice (Student’s *t*-test, t = 0.47, p = 0.64) and during the time of egg incubation (Mann - Whitney *U* test, Z = −1.597, p = 0.11).

All measurements of illumination were taken by using lux meter LS-100 (Sonopan®, Poland) with accuracy 0.001 lux. Each internal light measurement was preceded by the measurement of the ambient light entering the nest box through the entrance hole. These measurements were performed at the nest box entrance hole with a head of the lux meter was pointed perpendicularly to the nest box front wall. There was no difference in the illumination at the entrance between dark and bright nest boxes (Mann – Whitney *U* test, Z = −0.015, n = 95, p = 0.988).

We analyzed two measures of light intensity inside the boxes: the absolute illumination, hereafter illumination and the percentage of ambient light that reaches the interior of the nest box, hereafter light transfer.

### Data analysis

We examined the normality of data distribution using Shapiro-Wilk tests. When data was not normally distributed, non-parametric tests were applied. In the nest site choice experiment, the binomial dependent variable was ‘nest box occupation’ (1 – occupied, 0 – not occupied) and was tested by using logistic regression model (LOGISTIC). In this model we used two binomial explanatory variables ‘type of nest box’ (1 – bright, 0 – dark) and ‘occupation in 2015’ (1 – occupied in 2015, 0 – not occupied in 2015). We also used one categorical independent variable, which was ‘study site’ (0 – site I, 1 – site II, 2 – site III). In the logistic regression model, categorical variables with more than two levels were recoded into binominal dummy variables^63^. In this procedure “study site I” was used as baseline, which means that each other study site (II and III) was compared in the model with “study site I”. Model fit was tested with Omnibus Tests of Model Coefficients (*p* < 0.05 indicates good fit) and Hosmer and Lemenshow Tests (*p* > 0.05 indicates good fit)^64^.

To analyze the variation in nest height and the sequence of nest box occupation, we used type III General Linear Model (GLM) with type of nest box (bright or dark) and study site (I, II or III) as the grouping factors. In the nest height model, we used clutch size and the date of first egg lay as covariates. Both factors are often related to an individuals’ quality in birds, i.e. superior individuals breeds earlier and lay bigger clutches^65–67^. On the other hand, nest size may reflect individual quality^49^. Therefore, we controlled the potential effect of clutch size and the date of first egg lay on the nest height variation. In the sequence of the nest box occupation model, we used the date of first egg lay (converted to Julian date) as a dependent variable. In both models the interaction terms were removed when non-significant. All tests were two-tailed and the alpha level for significant differences was set to 0.05. All statistical analyses were conducted in SPSS Statistics (v.23, IBM). Descriptive statistics were presented as a mean ± standard deviation or as a median (Me) with the first and the third quartile (Q_25–75%_).

All methods were approved by the Local Ethical Committee and State Office for Environment Protection and performed in accordance with Polish law.

## References

[1] Oishi T (2001). Multiphotoreceptor and multioscillator system in avian circadian organization. Microsc. Res. Tech..

[2] Underwood H, Steele CT, Zivkovic B (2001). Circadian organization and the role of the pineal in birds. Microsc. Res. Tech..

[3] Stapput K, Güntürkün O, Hoffmann KP, Wiltschko R, Wiltschko W (2010). Magnetoreception of directional information in birds requires nondegraded vision. Curr. Biol..

[4] Kempenaers B, Borgström P, Loës P, Schlicht E, Valcu M (2010). Artificial night lighting affects dawn song, extra-pair siring success, and lay date in songbirds. Curr. Biol..

[5] Dominoni D, Quetting M, Partecke J (2013). Artificial light at night advances avian reproductive physiology. Proc. Biol. Sci.

[6] Shanawany MM (1982). The effect of ahemeral light and dark cycles on the performance of laying hens. A review. World’s Poult. Sci. J..

[7] Shanawany MM (1983). Sexual maturity and subsequent laying performance of fowls under normal photoperiods. A review 1950–1975. World’s Poult. Sci. J..

[8] Ernst RA, Millam JR, Mather FB (1987). Review of life-history lighting programs for commercial laying fowls. World’s Poult. Sci. J..

[9] Min JK (2012). Effect of monochromatic light on sexual maturity, production performance and egg quality of laying hens. Avian Biol. Res..

[10] Rowan W (1925). Relation of light to bird migration and developmental changes. Nature.

[11] Maurer G (2015). First light for avian embryos: eggshell thickness and pigmentation mediate variation in development and UV exposure in wild bird eggs. Funct. Ecol..

[12] Buschmann JUF, Manns M, Güntürkün O (2006). ‘Let there be light!’ pigeon eggs are regularly exposed to light during breeding. Behav. Processes.

[13] Maurer G, Portugal SJ, Cassey P (2011). Review: an embryo’s eye view of avian eggshell pigmentation. J. Avian Biol..

[14] Cooper CB, Voss MA, Ardia DR, Austin SH, Robinson WD (2011). Light increases the rate of embryonic development: implications for latitudinal trends in incubation period. Funct. Ecol.

[15] Austin SH, Hau M, Robinson WD (2014). Effect of photoperiod on incubation period in a wild passerine. Sylvia atricapilla. J. Avian Biol..

[16] Shutze JV, Lauber KJ, Kato M, Wilson WO (1962). Influence of incandescent and coloured light on chicken embryos during incubation. Nature.

[17] Isakson TS, Huffman JB, Siegel PB (1970). Intensities of incandescent light and development of chick embryos in-ovo and *in-vitro*. Comp. Biochem. Physiol.

[18] Lauber KJ (1975). Photoacceleration of avian embryogenesis. Comp. Biochem. Physiol. A Comp. Physiol.

[19] Fairchild BD, Christensen VL (2000). Photostimulation of turkey eggs accelerates hatching times without affecting hatchability, liver or heart growth, or glycogen content. Poult. Sci..

[20] Ghatpande A, Ghatpande S, Khan M (1995). Effect of different intensities of fluorescent light on the early development of chick embryos in ovo. J. Cell. Mol. Biol. Res..

[21] Robbins KR, Adekunmisi AA, Shirley HV (1984). The effect of light regime on growth and pattern of body fat accretion of broiler chickens. Growth.

[22] Lewis PD, Gous RM (2009). Responses of poultry to ultraviolet radiation. World’s Poult. Sci. J..

[23] Wesołowski T, Maziarz M (2012). Dark tree cavities - a challenge for hole nesting birds?. J. Avian Biol..

[24] Maziarz M, Wesołowski T (2014). Does darkness limit the use of tree cavities for nesting by birds?. J. Ornithol..

[25] Raap T, Pinxten R, Eens M (2016). Artificial light at night disrupts sleep in female great tits (*Parus major*) during the nestling period, and is followed by a sleep rebound. Environ. Pollut..

[26] Raap T, Pinxten R, Eens M (2015). Light pollution disrupts sleep in free-living animals. Sci. Rep.

[27] Marasco, V. & Spencer, K. A. Improvements in our understanding of behavior during incubation in Nests, eggs, and incubation (ed. Deeming, D. C. & Reynolds, S. J.) 142–150 (Oxford University Press, 2015).

[28] Maziarz M, Wesołowski T, Hebda G, Cholewa M (2015). Natural nest-sites of great tits (*Parus major*) in a primeval temperate forest (Białowieża National Park, Poland). J. Ornithol..

[29] Appelby MC, McRae HE, Peitz BE (1984). The effect of light on the choice of nests by domestic hens. Appl. Anim. Ethol.

[30] Lumsden HG (1976). Choice of nest boxes by starlings. Wilson Bull..

[31] Lumsden HG, Page RE, Gauthier M (1980). Choice of nest boxes by common goldeneyes in Ontario. Wilson Bull..

[32] Rohrbaugh RW, Yahner RH (1997). Effects of macrohabitat and microhabitat on nest-box use and nesting success of American kestrels. Wilson Bull..

[33] Blagosklonov KN (1970). On the importance of illumination in the nests of birds nesting in tree hollows. Bull. Mosc. Soc. Natur. Biol. Ser..

[34] Pitts DT (1977). Do eastern bluebirds and house sparrows prefer nest boxes with white or black interiors?. Bird-Banding.

[35] Lumsden HG (1986). Choice of nest boxes by tree swallows, *Tachycienta bicolor*, house wrens, *Troglodytes aedon*, eastern bluebirds, *Sialia sialis*, and European starlings, *Sturnus vulgaris*. Can. Field. Nat..

[36] Cassey P (2009). Biological Optics: seeing colours in the dark. Curr. Biol..

[37] Alatalo, R. V., Lundberg, A. & Ulfstrand, S. Habitat selection in the pied flycatcher *Ficedula hypoleuca* in: Habitat selection in birds (ed. Cody, M. L.) 59–83 (Academic Press, Inc., 1985).

[38] Ekner-Grzyb A, Żołnierowicz KM, Lisicki D, Tobółka M (2015). Habitat selection taking nest-box age into account: a field experiment in secondary hole-nesting birds. Folia Zool..

[39] Mänd R, Tilgar V, Lõhmus A, Leivits A (2005). Providing nest boxes for hole-nesting birds - Does habitat matter?. Biodivers. Conserv..

[40] Keller LF, van Noordwijk AJ (1994). Effects of local environmental conditions on nestling growth in the great tit *Parus major* L. Ardea.

[41] van Noordwijk AJ, McCleery RH, Perrins CM (1995). Selection for the timing of great tit breeding in relation to caterpillar growth and temperature. J. Anim. Ecol..

[42] Tomás G (2006). Nest weight and female health in blue tit (*Cyanistes caeruleus*). Auk.

[43] Mainwaring MC, Hartley IR (2009). Experimental evidence for state-dependent nest weight in the blue tit. Cyanistes caeruleus. Behav. Process..

[44] Broggi J, Senar JC (2009). Brighter great tit parents build bigger nests. Ibis.

[45] Wesołowski T (2002). Anti-predator adaptations in nesting marsh tits *Parus palustris*: the role of nest-site security. Ibis.

[46] Alabrudzińska J (2003). Effects of nest characteristics on breeding success of great tits *Parus major*. Acta Ornithol..

[47] Wesołowski T, Czeszczewik D, Rowiński P, Walankiewicz W (2002). Nest soaking in natural holes - a serious cause of breeding failure?. Ornis Fenn.

[48] Bańbura J (2001). Sex differences in parental care in a Corsican blue tit *Parus caeruleus* population. Ardea.

[49] Álvarez E, Barba E (2008). Nest quality in relation to adult bird condition and its impact on reproduction in great tits *Parus major*. Acta Ornithol..

[50] Møller AP (2014). Variation in clutch size in relation to nest size in birds. Ecol. Evol.

[51] Glądalski M (2015). Inter-annual and inter-habitat variation in breeding performance of blue tits (*Cyanistes caeruleus*) in central Poland. Ornis Fenn.

[52] Lambrechts MM (2016). Nest size is not closely related to breeding success in blue tits: a long-term nest-box study in a Mediterranean oak habitat. Auk.

[53] Lambrechts MM, Blondel J, de Franceschi C (2016). Nest size is positively correlated with fledging success in Corsican blue tits (*Cyanistes caeruleus*) in an insular oak-dominated habitat mosaic. J. Ornithol..

[54] Mainwaring MC, Hartley IR, Lambrechts MM, Deeming DC (2014). The design and function of birds’ nests. Ecol. Evol.

[55] Lima SL (2009). Predators and the breeding birds: behavioral and reproductive flexibility under the risk of predation. Biol. Rev.

[56] Mazgajski TD, Rykowska Z (2008). Dependence of nest mass on nest hole depth in the great tit *Parus major*. Acta Ornithol..

[57] Kaliński A (2014). Does the threat of European Pine Marten (*Martes martes*) predation influence the height of nests built by blue tits (*Cyanistes caeruleus*) and great tits (*Parus major*)?. Avian Biol. Res..

[58] Götmark F, Ahlström M (1997). Parental preference for red mouth of chicks in a songbird. Proc. R. Soc. B Biol. Sci.

[59] Munoz AR, Altamirano M, Takasu F, Nakamura H (2007). Nest light environment and the potential risk of common cuckoo (*Cuculus canorus*) parasitism. Auk.

[60] Honza M, Procházka P, Morongová K, Čapek M, Jelínek V (2011). Do nest light conditions affect rejection of parasitic eggs? A test of the light environment hypothesis. Ethology.

[61] Hansell, M. H. Standardising the nest description in Bird Nests and Construction *Behaviour* (ed. Hansell, M. H.) 42–43 (Cambridge University Press, 2000).

[62] Lessells CM, Boag PT (1987). Unrepeatable repeatabilities: a common mistake. Auk.

[63] Field, A. Logistic Regression in Discovering statistics using *SPSS* (ed. Field, A.) 264–297 (Oriental Press, 2009).

[64] Pallant, J. F. Logistic reggresion in SPSS Survival Manual: a step by step guide to data analysis using *SPSS* (ed. Pallant, J. F.) 160–171 (Allen & Unwin, 2005).

[65] Rowe L, Ludwig D, Schluter D (1994). Time, Condition and the seasonal decline of avian clutch size. Am. Nat..

[66] Møller AP (1994). Phenotype-dependent arrival time and its consequences in a migratory bird. Behav. Ecol. Sociobiol..

[67] Descamps S, Bêty J, Love OP, Gilchrist HG (2011). Individual optimization of reproduction in a long-lived migratory bird: a test of the condition-dependent model of laying date and clutch size. Funct. Ecol.

